# Evaluation of transorbital sonography measures of optic nerve diameter in the context of global and regional brain volume in multiple sclerosis

**DOI:** 10.1038/s41598-023-31706-5

**Published:** 2023-04-05

**Authors:** Szabolcs István Antal, Bálint Kincses, Dániel Veréb, András Király, Eszter Tóth, Bence Bozsik, Péter Faragó, Nikoletta Szabó, Krisztián Kocsis, Krisztina Bencsik, Péter Klivényi, Zsigmond Tamás Kincses

**Affiliations:** 1 Department of Radiology, Albert Szent-Györgyi Clinical Center, University of Szeged, Szeged, Hungary; 2 Department of Psychiatry, Albert Szent-Györgyi Clinical Center, University of Szeged, Szeged, Hungary; 3 Institute of Diagnostic and Interventional Radiology and Neuroradiology, University Hospital Essen, Essen, Germany; 4 Department of Neurology, Albert Szent-Györgyi Clinical Center, University of Szeged, Szeged, Hungary; 5 Department of Neurobiology, Care Sciences and Society, Karolinska Institutet, Stockholm, Sweden

**Keywords:** Multiple sclerosis, Multiple sclerosis, Diagnostic markers, Image processing, Imaging techniques

## Abstract

Transorbital sonography (TOS) could be a swift and convenient method to detect the atrophy of the optic nerve, possibly providing a marker that might reflect other quantitative structural markers of multiple sclerosis (MS). Here we evaluate the utility of TOS as a complementary tool for assessing optic nerve atrophy, and investigate how TOS-derived measures correspond to volumetric brain markers in MS. We recruited 25 healthy controls (HC) and 45 patients with relapsing–remitting MS and performed B-mode ultrasonographic examination of the optic nerve. Patients additionally underwent MRI scans to obtain T1-weighted, FLAIR and STIR images. Optic nerve diameters (OND) were compared between HC, MS patients with and without history of optic neuritis (non-ON) using a mixed-effects ANOVA model. The relationship between within-subject-average OND and global and regional brain volumetric measures was investigated using FSL SIENAX, voxel-based morphometry and FSL FIRST. OND was significantly different between HC-MS (HC = 3.2 ± 0.4 mm, MS = 3 ± 0.4 mm; *p* < *0.019*) and we found significant correlation between average OND and normalised whole brain (β = 0.42, *p* < 0.005), grey matter (β = 0.33, *p* < 0.035), white matter (β = 0.38, *p* < 0.012) and ventricular cerebrospinal fluid volume (β = − 0.36, *p* < 0.021) in the MS group. History of ON had no impact on the association between OND and volumetric data. In conclusion, OND is a promising surrogate marker in MS, that can be simply and reliably measured using TOS, and its derived measures correspond to brain volumetric measures. It should be further explored in larger and longitudinal studies.

## Introduction

Multiple sclerosis (MS) is a chronic autoimmune inflammatory disease of the central nervous system (CNS). It is characterized by acute demyelinating episodes and chronic axonal loss, which might lead to irreversible neurological and cognitive deficit^1^. The visual pathway, especially the optic nerve, is not only commonly involved in the disease but is also one of the first sites where inflammation develops^2^. The symptoms of optic nerve inflammation—optic neuritis (ON)—may include painful eye movements, followed by unilateral visual dysfunction^2^. The prognosis is often favourable even without treatment^3,4^. Optic neuritis is reported to be the first manifestation of the disease in about one third of the patients and around 70% of patients experience ON symptoms^5–7^. Moreover, a long-term prospective study has shown that 74% of women and 34% of men who had been previously affected by ON will have been diagnosed with MS within 15 years^8^. Furthermore it was shown that optic nerve function is altered even in the absence of optic neuritis in MS patients^9^.

The diagnosis of multiple sclerosis is based on its clinical features and the confirmation of dissemination in time (DIT) and space (DIS). From a radiological point of view, DIS can be proved by lesions in certain pre-determined locations (periventricular, (juxta)cortical, infratentorial, spinal). In recent years there has been an ongoing debate whether optic nerve lesions would contribute to the DIS criterion. While the 2016 MAGNIMS criteria^10^ suggested to include the optic nerve lesions to DIS criteria, the 2017 revision of the McDonald criteria^11^ did not consider optic nerve lesions of dysfunction for DIS due to insufficient evidence supporting it. The 2021 MAGNIMS consensus^12^ also suggests to use dedicated optic nerve MRI only optionally in the diagnostic work-up.

The choice of modality for detecting structural abnormalities associated with the optic nerve is a matter of debate, there are several candidates. Optical coherence tomography (OCT) is a method based on interferometry which uses low coherence infrared light to depict light-scattering objects in high resolution in two dimensions. It has high intra- and interobserver reproducibility^13,14^, and it is used extensively in the examination of the anterior visual pathway. OCT has proven to be reliable in detecting the atrophy of retinal fibres, which correlates well with axonal loss^15^, brain atrophy^16–18^ as well as the degree of disability^17,19–21^ and visual impairment^22–24^ in MS patients^15,20,25–29^. Furthermore, previous studies have shown that there is close correlation between retinal nerve fibre layer (RNFL) thickness and optic nerve diameter suggesting that by measuring OND, one can indirectly assess axonal loss as well^30,31^.

Transorbital ultrasonography (TOS), is a promising, non-invasive bedside technique to examine the optic nerve. Ultrasonographic measurements of the optic nerve sheath diameter (ONSD) correlate well with the MRI measurements taken at the same distance from the optic disc (3 and 5 mm)^32^. Furthermore, TOS has high intra- and interobserver reliability, while also being an inexpensive method that can be easily mastered by any clinician^33,34^. Its use is widespread in the evaluation of intracranial hypertension by measuring the optic nerve sheath diameter (ONSD), for which it has proven to be useful^35–37^. TOS has proven viable in diagnosing MS as well: by measuring optic nerve diameter (OND) or optic nerve sheath diameter (ONSD), atrophy^38–40^ or inflammation^41^ of the nerve can be revealed, which can be a first manifestation of the disease.

Earlier studies showed that inflammation of the anterior visual system reaches the posterior system as well via retrograde trans-synaptic axonal degeneration^42,43^. According to this—in theory—it is possible to identify biomarkers among the anterior visual structures—such as optic nerve diameter (OND), which can be easily measured with ultrasonography—that represent other global quantitative markers—such as brain atrophy and regional grey matter volumes. Proof to this theory would mean that by measuring optic nerve diameter, one could indirectly assess global brain parameters as well. Studies that investigate this association between optic nerve parameters and conventional MS-related markers report conflicting results and are few in number, mainly focusing on the relationship between the optic nerve *sheath* diameter, disease parameters and altered brain structure^39^. Bare optic nerve diameter, however, might be more representative of axonal loss, therefore it might reflect structural brain alterations more accurately^15^.

It is unclear whether the structural changes of the optic nerve translate to brain atrophy, or they develop independently to each other as a result of the diffuse nature of MS. Since global brain atrophy and structural alterations of the optic nerve appear early of the disease^2^ and continue throughout the disease course, we hypothesize that OND correlates with global and regional brain volumes. In this cross-sectional study we first investigate if OND differs between MS patients and healthy individuals. Then, the relationship between OND and global and regional structural MRI markers in MS patients is also examined. Finally, we assess how the previous occurrence of optic neuritis influences the correlation between OND and the aforementioned structural MRI markers.

## Materials and methods

### Participants

We recruited 45 patients aged between 18 and 50 years diagnosed with relapsing–remitting multiple sclerosis (RRMS) according to the 2005, 2010 or the 2017 McDonald criteria depending on the time of their diagnosis^11,44,45^. All of them were enlisted from the Multiple Sclerosis Outpatient Clinic of the Department of Neurology, University of Szeged. RRMS patients were only included if they had been relapse-free for three months prior to the time of examination and received disease-modifying therapy. Among exclusion criteria for patients were current ophthalmological conditions (e.g. glaucoma), neurological diseases other than RRMS, as well as any psychiatric diseases and untreated diabetes or hypertension. As control group, we recruited 25 age- and gender-matched healthy volunteers. Exclusion criteria for controls were any neurological, psychiatric or ophthalmological disease, as well as untreated diabetes or hypertension. RRMS patients underwent neurological examination to determine their degree of disability according to the Expanded Disability Status Scale (EDSS)^46^. Detailed demographic data of participants are described in Table 1.

**Table 1:** Demographic data of participants.

	Healthy controls	RRMS patients
# of subjects (# of eyes)	25 (50)	45 (90)
Females	13	31
Age (years)	37.72 ± 14.8 (range: 21.6—60.4)	40.97 ± 9.8 (range: 22.5–58.5)
Disease duration (years)	–	8.945 ± 5.88 (range: 0.4–24)
EDSS	–	1.211 ± 1.094 (range: 0—4)
Treatment regimen	–	GA—29% Te—22% F—22% A—9% IFNb—7% DF—7% N—2% P—2%

Mean ± standard deviation (SD) are described. Range intervals are as follows: age: 21.6–60.4 years for controls, 22.5–58.5 years for patients; disease duration: 0.4–24 years; EDSS: 0–4. Treatment regimen abbreviations: *GA* glatiramer-acetate, *Te* teriflunomide, *F* fingolimod, *A* alemtuzumab, *IFNb* interferon beta 1a, *DF* dimethyl fumarate, *N* natalizumab, *P* pramipexole.

All participants provided their written informed consent according to the Declaration of Helsinki and the Regional and Institutional Human Biomedical Research Ethics Committee of University of Szeged, Szeged, Hungary approved all experimental protocols (000002/2016/OTIG). All research was performed in accordance with relevant guidelines and regulations.

### Image acquisition and classification

#### Ultrasound

**Figure 1:**
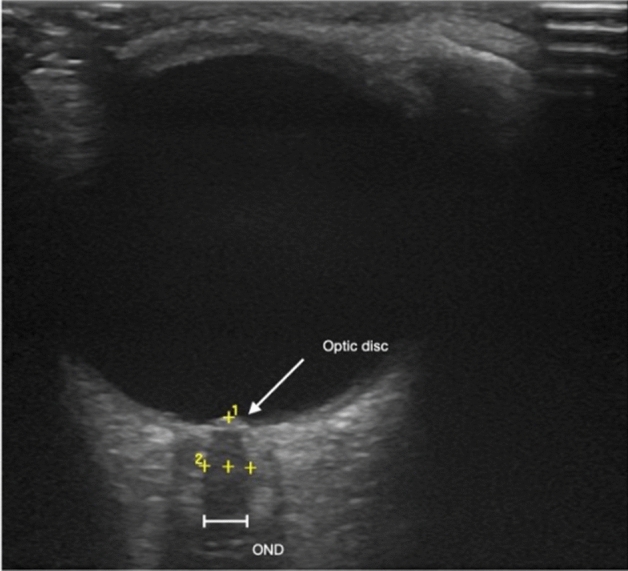
Example measurement of optic nerve diameter with transorbital ultrasonography. Optic nerve diameters were measured at a 3 mm distance behind the optic disc in a longitudinal view of the nerve.

B-mode ultrasonic measurement was performed at the site of outpatient examination using the 12 MHz linear transducer of a GE Logiq P9 ultrasound system. The transducer was placed on the patients’ eyelid horizontally, with proper lubrication applied. The mechanical index was reduced below 0.4 and the duration of the examination was limited to a maximum of 3 min per eye to avoid thermal damage. Cautious scanning of the orbita was performed in the horizontal plane to identify the optic nerve and the part that appeared widest upon visual assessment was used for measurement. Measurements were taken at a 3 mm distance from the optic disc in line with the longitudinal axis as described in previous studies^37,38,47^. The inner hypoechogenic area in the longitudinal section was identified as the optic nerve and the outer hyperechogenic area as the optic nerve sheath. We measured the diameter of the inner hypoechogenic area (Fig. 1). The average time gap between the latest documented occurrence of optic neuritis and transorbital sonography was 40 ± 37.141 months for ON patients.

#### MRI acquisition

Patients underwent MRI measurements on a 3T GE MR750W Discovery scanner (GE, Milwaukee, USA) at the Department of Radiology, University of Szeged. For every patient the following images were acquired according to our recent recommendations^48,49^. 3D axial fast spoiled gradient echo (FSPGR) T1-weighted images (TR = 450 ms, TE = 4.2 ms, FOV = 256 mm, slice thickness 1 mm, flip angle 12), 2D spin-echo (SE) T1-weighted images (TR = 500 ms, TE = 4.2 s, FOV = 240 mm, slice thickness 1.4 mm, flip angle 73), 3D sagittal fluid-attenuated inversion recovery (FLAIR) (TR = 6.7 ms, TI = 1.8 ms, FOV = 250 ms, slice thickness 1.4 mm), and 3D double inversion recovery images (DIR) (TR = 7000 ms, TE = 90 ms, TI = 546 ms, TI2 = 2900 ms, FOV = 250 mm, slice thickness 1.4 mm), 2D axial T2 and proton density (PD) weighted dual echo fast spin echo sequences (TR = 3000 ms, TE = Min Full, TE2 = 102 ms, FOV = 240 mm, flip angle 125, slice thickness 3.0 mm), and 2D coronal short tau inversion recovery (STIR) images on the optic nerve and the chiasm (TR = 3000 ms, TE = 42 ms, TI = 185 ms, FOV = 240 mm, flip angle 111). All the MRI images were evaluated by a single neuroradiologist (ZTK) to reduce the interrater variability^50^. We used 3D T1 weighted sequences for volumetric analyses, sagittal FLAIR to detect periventricular and juxtacortical lesions perpendicular to the corpus callosum, and 3D DIR sequence to detect (juxta-)cortical lesions. The coronal STIR images were used to identify optic nerve lesions, but all other sequences were made available if doubt. The median time gap between MRI and TOS measurements was 139 ± 77 days.

#### Patients’ group classification

After the imaging procedure, we classified the MS patients’ eye parameters into groups according to the presence of optic nerve lesions on MRI and history of optic neuritis. Medical history, functional (VEP and/or visual acuity) and structural data (MRI) were extracted from medical records retrospectively to determine prior optic neuritis. The detailed classification is depicted in Table 2.

**Table 2:** Classification of patients.

	Patients		Eyes	
HC	25		50	
MS	45		90	
ON +	23		31	
	MR +: 17	MR −: 6	MR +: 18	MR −: 13
ON −	22		59	
	MR +: 8	MR −: 14	MR +: 13	MR −: 46
MR +	25		31	
MR −	20		59	

We created four groups within the patients: two based on the clinical history of optic neuritis (ON + and ON −), and two based on the presence of optic lesions detectable by MRI (MR + and MR −). The ‘Patients’ column containts sample sizes of pooled groups i.e. where the mean of the two optic nerves was used, whereas the ‘Eyes’ column containts sample sizes achieved by using the optic nerve diameters individually. Among both groups based on history of optic neuritis, subgroups were created according to the presence optic lesions. (e.g. 13 of the total 59 eyes without optic neuritis had asymptomatic optic nerve lesions).

### Image analysis

#### Cortical and subcortical volumetry

To carry out the brain volumetry analysis, we used the FMRIB Software Library (FSL v5.0.10^51^). Cross-sectional estimates of global and partial brain tissue volumes, normalised for individual head size, were acquired using FSL SIENAX^52,53^. During the course of this analysis, skull and non-brain images are extracted and an affine registration applied to MNI152 standard space takes place to determine the volumetric scaling factor used in the normalisation step. Finally, tissue-type segmentation is performed to obtain separate estimates for grey matter (GM), cortical (or peripheral) grey matter (pGM), white matter (WM) and ventricular CSF (vCSF) volume^54^. From the T2 FLAIR images binary lesion masks were created manually using FSLeyes^55^, which were then reviewed by two experienced (and blinded) neuroradiologists independently. Since lesions can influence segmentation and, consequently, volume estimation, FSL’s *lesion_filling*^56^ tool was used before the analysis. The volumetric data of subcortical structures were estimated using FSL FIRST, a model-based segmentation and registration tool, which uses deformable shape models based on previous training data to obtain an optimal shape fit for a range of subcortical structures^53^; here we focused on the thalami given their frequently altered structure and relevance to MS-related pathologic processes described in previous studies^57^. The volumetric data of subcortical structures were also normalised for individual head size by multiplication with the volumetric scaling factor obtained during the SIENAX analysis. To investigate the relationship between cortical and subcortical volumetric measures and average OND, we calculated partial Pearson’s correlation, correcting for age and sex, which reportedly influence cortical and subcortical structure volumes^58,59^. Furthermore, we employed Student’s independent samples T-tests to assess differences in volumetric measures between ON and non-ON MS patients.

#### Regional grey matter changes

Associations between average OND and regional cortical grey matter density were investigated with an optimised protocol of voxel-based morphometry (VBM)^60^, using the FSL implementation^61^. T1-weighted images were brain-extracted and grey matter-segmented, then normalised to MNI152-space using non-linear registration with FNIRT to create a study-specific template. Individual images were then registered to this template and smoothed using an isotropic Gaussian kernel with a sigma of 3 mm. FSL’s *lesion_filling* was applied before the analysis. In the statistical analysis we also applied a standard GM mask to the output of VBM to minimise the inclusion of any possible intensity differences of the white matter. Statistical inference was performed using a general linear model (GLM) based approach implemented in FSL’s randomise, with threshold-free cluster enhancement to account for spatial interdependence and correcting for multiple comparisons by controlling the family-wise error^62^. We assessed the following designs in the GLM framework: correlation between average OND and voxelwise measures in the whole MS group; difference of the regression slope between average OND and voxelwise measures in ON and non-ON patients (continuous covariate interaction). We included age, sex and the time between MRI scans and TOS measurements as nuisance regressors. For the VBM analyses, we also tested designs that further included the volumetric scaling factor in accordance with previous studies^42,43^.

### Statistical analysis

Statistical analysis was carried out using RStudio version 1.2^63^. The *car*^64^ and *lme4*^65^ packages were used for statistical evaluation and model building. *ggplot2*^66^ was used for visualisation. To evaluate correlation between optic nerve diameter, and clinical, as well as volumetric parameters, a linear mixed effects model was used. In the model the subject was the random effect and the groups (HC-MS, ON–non-ON), age, gender, normalised brain- and lesion volume, and lesion count were handled as separate fixed effects. ANOVA was calculated from the model to evaluate variability between groups. The eyes of the patients were inspected independently because an average of the two eyes could be misleading and the difference within a single patient—regarding laterality and involvement in optic neuritis—is negligible according to previous studies^39,67^. However, since we used average OND as the independent variable in the MRI analysis, we also tested whether average OND differs between HC and the pooled MS group, and patients with and without history of ON using independent samples T-tests. Significance level was set at α = 0.05. We also assessed whether disease duration, EDSS and total lesion volume correlate with average OND. No statistical corrections for multiple comparisons have been made.

## Results

### Demographical data

Patients with (n = 23) and without a history of optic neuritis (n = 22) did not differ in terms of disease duration, lesion load (Student’s t-test; *p* < 0.26 and *p* < 0.23) or EDSS (Mann–Whitney U-test; *p* < 0.8). Healthy participants (n = 25) did not differ from the MS group (n = 45) in terms of age (Student’s t-test; *p* < 0.3) and sex distribution (Fisher’s exact test; *p* < 0.2). There was no difference in age (Student’s t-test; *p* < 0.825) and sex distribution (Fisher’s exact test; *p* < 0.212) between the ON (n = 23) and NON (n = 22) groups either. While not tested formally at the time of the TOS the visual acuity of the patients was collected retrospectively from their clinical charts and all patients had a visual acuity of 1 or corrected to 1.

### Diameter difference

**Figure 2:**
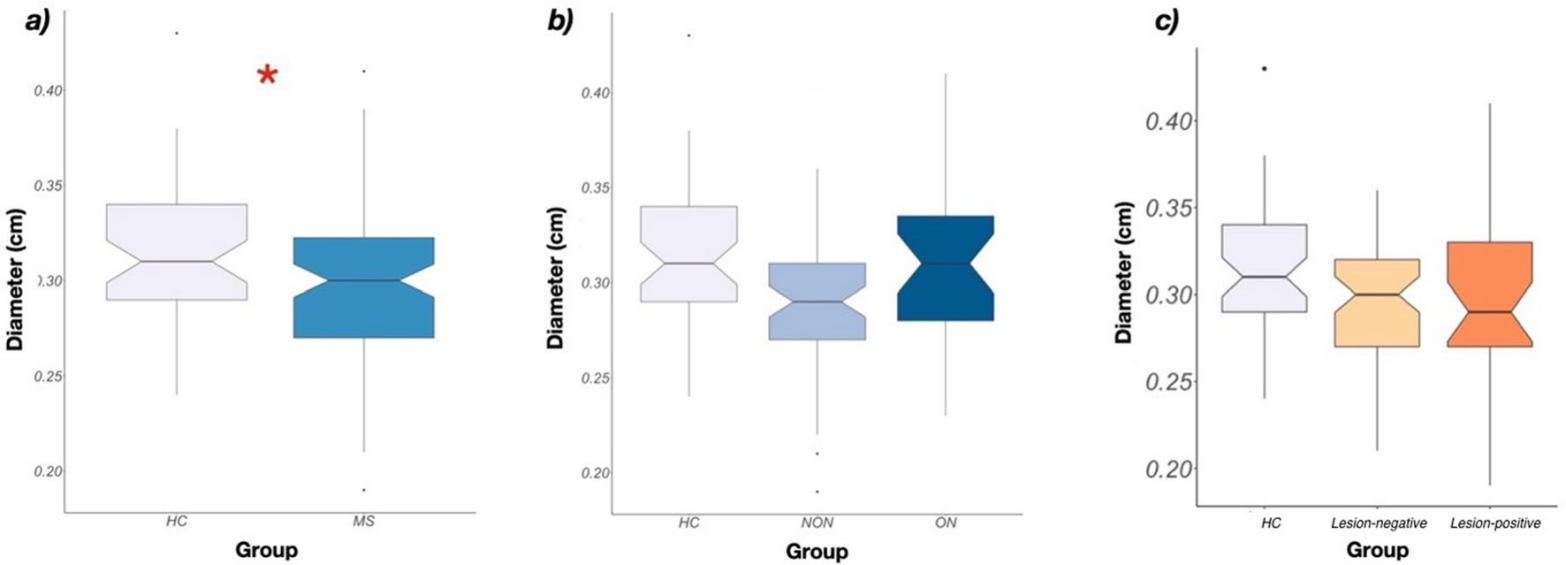
Difference of OND between groups. (**a**) Diameter difference between the HC and MS groups. The diameter of the optic nerve in MS patients was significantly smaller than that of the healthy controls (HC = 3.174 ± 0.376 mm, n = 50; MS = 2.968 ± 0.406 mm, n = 90; *p* < 0.0178). (**b**) Diameter difference between the HC, ON and NON groups. The diameters in the ON-NON groups did not differ significantly (ON = 3.113 ± 0.422 mm, n = 31; NON = 2.901 ± 0.384 mm, n = 59; *p* < 0.089). (**c**) Diameter difference between the optic nerves that had MRI-detectable lesions within them (Lesion-positive, n = 31) and those that had not (Lesion-negative, n = 59). We found no significant difference between the the two groups.

The diameter of the optic nerve (OND) in MS patients was significantly smaller than that of the healthy controls (HC = 3.174 ± 0.376 mm, n = 50; MS = 2.974 ± 0.407 mm, n = 90; *p* < *0.0178*) (Fig. 2a). There was no significant difference of OND between the ON and NON groups (ON = 3.113 ± 0.422 mm, n = 31; NON = 2.901 ± 0.384 mm, n = 59; *p* < 0.089) (Fig. 2b). We found no significant difference between the diameters of optic nerves that had MRI-detectable lesions within them (2.99 ± 0.482 mm, n = 31) and those that had not (2.959 ± 0.372 mm, n = 59) (Fig. 2c). Average OND was also smaller in the pooled MS group (n = 45) compared to HC (n = 25; *p* < 0.022) but did not differ between patients with (n = 23) and without history of ON (n = 22).

### Correlation between clinical parameters and OND

Neither disease duration (ON group: R = 0.06, *p* < 0.75, n = 31; non-ON group: R = 0.065, *p* < 0.062, n = 59), nor EDSS scores (ON group: R = 0.15, *p* < 0.42, n = 31; non-ON group: R = 0.015, *p* < 0.91, n = 59) have shown significant correlation with the diameter of the optic nerve in either group when considering the eyes individually, or when average OND was used (ON group: n = 23; NON group: n = 22).

### Correlation between global cortical and subcortical volumetric data and OND

Our analysis has showed that individual OND in the MS group (n = 90) correlated significantly with normalised total brain volume (β = 0.279; *p* < 0.007), and within the patients, the NON group (n = 59) showed significant correlation (β = 0.359; p < 0.009), after correcting for age, sex, head size (i.e. scaling factor), disease duration, EDSS and lesion volume. We also found significant correlation between average OND and normalised total brain volume (β = 0.421, *p* < 0.005), GM volume (β = 0.326, *p* < 0.035), WM volume (β = 0.379, *p* < 0.012) and vCSF volume (β = –0.357, *p* < 0.021) in the pooled MS group (n = 45), corrected for age, sex and the time between TOS and MRI measurements. The correlation remained significant for whole-brain and grey matter volume when average OND was normalised for head size with the volumetric scaling factor (total brain volume: β = 0.32, *p* < 0.039; GM volume: β = 0.322, *p* < 0.038). We found that lesion volume did not correlate significantly with optic nerve diameter. On the other hand, lesion count in the NON group (n = 59) correlated well with OND (β = –0.412; *p* < 0.002). No significant correlation has been revealed between OND subcortical structure volumes.

### Correlation between regional grey matter density and OND

We found no correlation between average OND and regional grey matter density in the VBM analysis (R = 0.14, *p* < 0.36).

### Impact of optic neuritis on the association between OND and grey matter atrophy

We found no interaction between average OND and ON–NON group differences (n = 23 and n = 22 respectively) in the VBM analysis, meaning there was no difference regarding regional grey matter change-OND regression slopes between ON and NON MS patients. Regarding global brain volumes, the association between average OND and SIENAX-derived parameters retained statistical significance when we also corrected for history of ON; in fact, the correlation became stronger (NBV: β = 0.47, *p* < 0.002; GM: β = 0.392, *p* < 0.011; WM: β = 0.449, *p* < 0.003; vCSF: β = − 0.36, *p* < 0.021).

## Discussion

In our study we showed that the optic nerve diameter (OND) was lower in RRMS patients compared to the healthy population, and that the optic nerve diameter correlated well with brain atrophy measures. No correlation was found between OND and regional grey matter volumes.

Over the years, numerous biomarkers underwent evaluation to see which might assist in the diagnostic process of MS or reliably predict various aspects of it—such as response to therapy, progression and prognosis. Axonal loss is one of the most important factors that contribute to irreversible disability in MS, therefore if it was possible to evaluate it, it could become a new useful biomarker of MS^19^. Studies provide evidence for trans-synaptic axonal degeneration in the visual pathway of MS patients^42,43^, suggesting that in theory, axonal loss and other global brain parameters could be indirectly estimated by examining the anterior visual system. However, the optimal examination method of optic structures is a matter of debate.

While OCT is a more precise method that can differentiate well between various layers of the retina, transorbital ultrasound (TOS) is only capable of roughly estimating the integrity of these layers, since it can only examine them as a whole. Despite of this, ultrasound still has advantages including its low skill requirement, high accessibility and high intra-and interobserver reliability^31,35,38,39,67^. Furthermore, it is also a viable method in assessing the condition of the optic nerve in acute inflammation as well as in chronic atrophy^40,41,68–71^.

In our study, we used B-mode ultrasonography to investigate how the optic nerve diameter varies across the MS and HC groups and found significantly smaller diameters in the MS group, which supports OND’s viability as a complementary marker of disease progression in MS. This confirms the results of recent studies^31,39,40^, in which the authors have also found the diameter to be smaller in MS using TOS measurements. We took our measurements in the longitudinal section and not in cross-section, because according to a previous study^38^, the cross-sectional measurement is not only unreliable, but also hard to accomplish properly. Other studies have shown that there is no significant difference between values measured at 3 mm and at 5 mm in MS. In our case, the measurements were taken at 3 mm behind the papilla. Based on our MRI examination, the group with lesions present in the optic nerve (lesion group) have not differed significantly from the group that had no lesions (non-lesion group). This could be attributed to the underestimation of optic nerve lesions, which is partly due to the relatively poor spatial resolution of the sequence used in the imaging of the nerve.

We found that average optic nerve diameter (OND) correlates with brain volumetric data in our sample. To our knowledge, this has not been investigated before; several studies examined associations between optic nerve sheath diameter (ONSD) and total/partial brain volumes,which are important measures in the monitoring of MS according to the clinical assessment of “no evidence of disease activity” (NEDA-4)^72^. However, we have not found correlation between ONSD and volumetric data. This might indicate that bare OND reflects brain volume loss more accurately. It also suggests that ultrasonographic measurement of the optic nerve diameter could augment the current MRI-based monitoring techniques. We could not replicate previous descriptions of bidirectional trans-synaptic degeneration in the visual pathway^42,43^; this suggests that the association between global and partial brain volume and OND might be attributed to the diffuse nature of the disease rather than visual system-specific alterations. Another possible explanation is that the patients in our study were in the early stages of the disease and the trans-synaptic degeneration could not reach detectable levels. One of these studies also reported that visual cortex thickness only correlated with anterior visual system alterations in patients with history of previous optic neuritis^43^. We directly investigated the effect of previous ON on the optic nerve diameter—brain volume association. There was no difference in the regression slopes in regional analyses and the correlation between OND and global brain volumes remained significant when we corrected for the history of optic neuritis (ON). These results indicate that previous history of ON has little or no influence on the association between average OND and brain volumetric measures.

The lack of correlation between OND and other disease parameters (disease duration, EDSS) was an unexpected result, since previous studies^15,31,38,39^ have shown an inverse correlation between these values, which is to be expected since atrophy is continuous during the course of the disease. Results are, however, conflicting: a study reported no associations between OND and disease duration but showed that OND and EDSS correlate^47^.

With our standardised TOS approach we examined only the most anterior part of the optic nerves. However, MS lesions are more common in the anterior part of the optic nerve than in the posterior part^73^, which might influence our optic nerve diameter measurements.

Our study is not without limitations. Since the patients included in our study were in good clinical condition, our analyses were focused on the lower end of the EDSS scale. Including other parts of the scale proportionally could result in finding correlation between clinical data and optic nerve diameter. The ultrasonographic measurements were taken at the time of the outpatient visits, when all included patients were asymptomatic for at least 3 months. In the case of patients with positive history of ON this indicates that at least 3 months have elapsed between the initial presentation of optic neuritis and the ultrasonographic measurement. Also, focal atrophy of the optic nerve has not been investigated on MRI scans, which could also influence our results. Another limitation is that although we have collected retrospective data regarding visual acuity, it was not measured during the outpatient visit, prior to ultrasonographic measurement. This could be improved in a future study, in which functional assessment of the visual system is performed (such as visual acuity and visual evoked potentials) alongside the structural evaluation. Moreover, the time gap between optic neuritis symptoms and ultrasonographic measurements were relatively long and showed great variability, which could further hinder the interpretation of our results. As mentioned earlier, improving the resolution of imaging sequences used to visualize optic nerve lesions could yield more accurate lesion count in the nerve and therefore more authentic correlation with other measurements. Further limitations include the relatively low number of participants and the cross-sectional design of our study. These can be improved in the future by larger and longitudinal studies.

## Conclusion

In conclusion, our findings confirm the results of previous studies that transorbital sonography is a valid paraclinical examination method able to detect the atrophy of the optic nerve in the early stages of MS. We also report an association between average optic nerve diameter and brain volumetric data, which combined with the results of previous studies, suggests that by measuring OND, one can indirectly estimate axonal loss and brain atrophy, therefore making OND a possible biomarker of disease activity, and TOS an additional viable and accessible bedside examination method.

## Data Availability

The datasets analysed and the code used during the current study are not publicly available due to containing personal information about the participants of the study; but they are available from the corresponding author upon reasonable request.

## References

[1] Waxman SG (2000). Multiple sclerosis as a neuronal disease. Arch. Neurol..

[2] Helmut W, Martin S (2015). Diagnostik und therapie der optikusneuritis. Dtsch. Arztebl. Int..

[3] Beck RW (1991). The clinical profile of optic neuritis: Experience of the optic neuritis treatment trial. Arch. Ophthalmol..

[4] Beck RW (1992). A randomized, controlled trial of corticosteroids in the treatment of acute optic neuritis. N. Engl. J. Med..

[5] Sørensen TL, Frederiksen JL, Brønnum-Hansen H, Petersen HC (1999). Optic neuritis as onset manifestation of multiple sclerosis: A nationwide, long-term survey. Neurology.

[6] Tintore M (2015). Defining high, medium and low impact prognostic factors for developing multiple sclerosis. Brain.

[7] Costello F (2013). The afferent visual pathway: Designing a structural-functional paradigm of multiple sclerosis. ISRN Neurol..

[8] Rizzo JF, Lessell S (1988). Risk of developing multiple sclerosis after uncomplicated optic neuritis: A long-term prospective study. Neurology.

[9] Felgueiras H (2016). Dyschromatopsia in multiple sclerosis patients: A marker of subclinical involvement?. J. Neuroophthalmol..

[10] Filippi M (2016). MRI criteria for the diagnosis of multiple sclerosis: MAGNIMS consensus guidelines. Lancet Neurol..

[11] Thompson AJ (2018). Diagnosis of multiple sclerosis: 2017 revisions of the McDonald criteria. Lancet Neurol..

[12] Wattjes MP (2021). 2021 MAGNIMS–CMSC–NAIMS consensus recommendations on the use of MRI in patients with multiple sclerosis. Lancet Neurol..

[13] Cettomai D (2008). Reproducibility of optical coherence tomography in multiple sclerosis. Arch Neurol..

[14] Syc SB (2010). Reproducibility of high-resolution optical coherence tomography in multiple sclerosis. Multiple Scler..

[15] Koraysha NA (2019). Evaluating optic nerve diameter as a possible biomarker for disability in patients with multiple sclerosis. Neuropsychiatr. Dis. Treat..

[16] Grazioli E (2008). Retinal nerve fiber layer thickness is associated with brain MRI outcomes in multiple sclerosis. J. Neurol. Sci..

[17] Siger M (2008). Optical coherence tomography in multiple sclerosis: Thickness of the retinal nerve fiber layer as a potential measure of axonal loss and brain atrophy. J. Neurol..

[18] Gordon-Lipkin E (2007). Retinal nerve fiber layer is associated with brain atrophy in multiple sclerosis. Neurology.

[19] Toledo J (2008). Retinal nerve fiber layer atrophy is associated with physical and cognitive disability in multiple sclerosis. Mult. Scler..

[20] Siepman TAM, Bettink-Remeijer MW, Hintzen RQ (2010). Retinal nerve fiber layer thickness in subgroups of multiple sclerosis, measured by optical coherence tomography and scanning laser polarimetry. J. Neurol..

[21] Sepulcre J (2007). Diagnostic accuracy of retinal abnormalities in predicting disease activity in MS. Neurology.

[22] Merle H (2008). Retinal peripapillary nerve fiber layer thickness in neuromyelitis optica. Invest. Ophthalmol. Vis. Sci..

[23] Noval S, Contreras I, Rebolleda G, Muñoz-Negrete FJ (2006). Optical coherence tomography versus automated perimetry for follow-up of optic neuritis. Acta Ophthalmol. Scand..

[24] Naismith RT (2009). Optical coherence tomography is less sensitive than visual evoked potentials in optic neuritis. Neurology.

[25] Petzold A (2010). Optical coherence tomography in multiple sclerosis: A systematic review and meta-analysis. Lancet Neurol..

[26] Abalo-Lojo JM (2014). Retinal nerve fiber layer thickness, brain atrophy, and disability in multiple sclerosis patients. J. Neuroophthalmol..

[27] Khalil D, Labib D (2015). Correlation between spectral-domain optical coherence tomography parameters and neurological functional disability in multiple sclerosis. J. Egypt. Ophthalmol. Soc..

[28] Saidha S (2015). Optical coherence tomography reflects brain atrophy in multiple sclerosis: A four-year study. Ann. Neurol..

[29] el Ayoubi NK (2016). Retinal measures correlate with cognitive and physical disability in early multiple sclerosis. J. Neurol..

[30] Lagrèze WA (2009). Retrobulbar optic nerve diameter measured by high-speed magnetic resonance imaging as a biomarker for axonal loss in glaucomatous optic atrophy. Invest. Ophthalmol. Vis. Sci..

[31] Pérez Sánchez S (2019). Usefulness of optic nerve ultrasound to predict clinical progression in multiple sclerosis. Neurología (English Edition).

[32] Bäuerle J (2013). Reproducibility and accuracy of optic nerve sheath diameter assessment using ultrasound compared to magnetic resonance imaging. BMC Neurol..

[33] Bäuerle J, Lochner P, Kaps M, Nedelmann M (2012). Intra- and interobsever reliability of sonographic assessment of the optic nerve sheath diameter in healthy adults. J. Neuroimaging.

[34] Ballantyne SA, O’Neill G, Hamilton R, Hollman AS (2002). Observer variation in the sonographic measurement of optic nerve sheath diameter in normal adults. Eur. J. Ultrasound.

[35] Bäuerle J, Nedelmann M (2011). Sonographic assessment of the optic nerve sheath in idiopathic intracranial hypertension. J. Neurol..

[36] Geeraerts T, Merceron S, Benhamou D, Vigué B, Duranteau J (2008). Non-invasive assessment of intracranial pressure using ocular sonography in neurocritical care patients. Intensive Care Med..

[37] Rajajee V, Fletcher JJ, Rochlen LR, Jacobs TL (2012). Comparison of accuracy of optic nerve ultrasound for the detection of intracranial hypertension in the setting of acutely fluctuating vs stable intracranial pressure: Post-hoc analysis of data from a prospective, blinded single center study. Crit. Care.

[38] Candeliere Merlicco A (2018). Transorbital ultrasonography for measuring optic nerve atrophy in multiple sclerosis. Acta Neurol. Scand..

[39] de Masi R (2016). Transbulbar B-mode sonography in multiple sclerosis: Clinical and biological relevance. Ultrasound Med. Biol..

[40] Carcelén-Gadea M (2019). Functional and structural changes in the visual pathway in multiple sclerosis. Brain Behav..

[41] Dees C, Buimer R, Dick AD, Atta HR (1995). Ultrasonographic investigation of optic neuritis. Eye (Basingstoke).

[42] Gabilondo I (2014). Trans-synaptic axonal degeneration in the visual pathway in multiple sclerosis. Ann. Neurol..

[43] Balk LJ (2015). Bidirectional trans-synaptic axonal degeneration in the visual pathway in multiple sclerosis. J. Neurol. Neurosurg. Psychiatry.

[44] Polman CH (2005). Diagnostic criteria for multiple sclerosis: 2005 revisions to the “McDonald Criteria”. Ann. Neurol..

[45] Polman CH (2011). Diagnostic criteria for multiple sclerosis: 2010 revisions to the McDonald criteria. Ann. Neurol..

[46] Kurtzke JF (1983). Rating neurologic impairment in multiple sclerosis: An expanded disability status scale (EDSS). Neurology.

[47] Raeesmohammadi L (2020). Transbulbar B-mode sonography in multiple sclerosis without optic neuritis; clinical relevance. Brain Res..

[48] Tóth E (2018). The role of MRI in measuring the effectivity of disease modifying treatments I. Ideggyogy Sz.

[49] Kincses ZT (2018). The role of MRI in measuring the effectivity of disease modifying treatments II. Ideggyogy Sz.

[50] Bozsik B (2022). Reproducibility of lesion count in various subregions on MRI scans in multiple sclerosis. Front. Neurol..

[51] Smith SM (2004). Advances in functional and structural MR image analysis and implementation as FSL. Neuroimage.

[52] Smith SM (2002). Accurate, robust, and automated longitudinal and cross-sectional brain change analysis. Neuroimage.

[53] Patenaude B, Smith SM, Kennedy DN, Jenkinson M (2011). A Bayesian model of shape and appearance for subcortical brain segmentation. Neuroimage.

[54] Zhang Y, Brady M, Smith S (2001). Segmentation of brain MR images through a hidden markov random field model and the expectation-maximization algorithm. IEEE Trans. Med. Imaging.

[55] FSLeyes|Zenodo. https://zenodo.org/record/7038115#.Y5sLb7LMLao.

[56] Battaglini M, Jenkinson M, de Stefano N (2012). Evaluating and reducing the impact of white matter lesions on brain volume measurements. Hum. Brain Mapp..

[57] Minagar A (2013). The thalamus and multiple sclerosis: Modern views on pathologic, imaging, and clinical aspects. Neurology.

[58] Király A (2016). Male brain ages faster: The age and gender dependence of subcortical volumes. Brain Imaging Behav..

[59] Ritchie SJ (2018). Sex differences in the adult human brain: Evidence from 5216 UK biobank participants. Cereb. Cortex.

[60] Good CD (2001). A voxel-based morphometric study of ageing in 465 normal adult human brains. Neuroimage.

[61] Douaud G (2007). Anatomically related grey and white matter abnormalities in adolescent-onset schizophrenia. Brain.

[62] Winkler AM, Ridgway GR, Webster MA, Smith SM, Nichols TE (2014). Permutation inference for the general linear model. Neuroimage.

[63] R Core Team. *R: A Language and Environment for Statistical Computing*. Preprint at (2018).

[64] Fox J, Weisberg S (2019). An R Companion to Applied Regression.

[65] Bates, D., Mächler, M., Bolker, B. & Walker, S. Fitting linear mixed-effects models using lme4. *J. Stat. Softw.* **67** (2015).

[66] Wickham H (2016). ggplot2: Elegant Graphics for Data Analysis.

[67] Lochner P (2014). Transorbital sonography in acute optic neuritis: A case-control study. Am. J. Neuroradiol..

[68] Fernández-Domínguez J, García-Rodríguez R, Mateos V (2012). Transorbital echography for assessment of optical nerve atrophy in demyelinating diseases: A pilot study. Rev. Neurol..

[69] Karami M, Janghorbani M, Dehghani A, Riahinejad M (2012). Orbital doppler evaluation of blood flow velocities in optic neuritis. Korean J. Ophthalmol..

[70] Dehghani A, Akhlaghi M, Salehi F, Giti M, Karami M (2012). Ultrasonography in distinguishing optic neuritis from nonarteritic anterior ischemic optic neuropathy. Adv. Biomed. Res..

[71] Lochner P (2016). B-mode transorbital ultrasononography for the diagnosis of acute optic neuritis: A systematic review. Clin. Neurophysiol..

[72] Kappos L (2016). Inclusion of brain volume loss in a revised measure of ‘no evidence of disease activity’ (NEDA-4) in relapsing-remitting multiple sclerosis. Mult. Scler..

[73] Mealy MA (2015). Longitudinally extensive optic neuritis as an MRI biomarker distinguishes neuromyelitis optica from multiple sclerosis. J. Neurol. Sci..

