# Author Correction: Evaluation of transorbital sonography measures of optic nerve diameter in the context of global and regional brain volume in multiple sclerosis

**DOI:** 10.1038/s41598-023-43994-y

**Published:** 2023-10-06

**Authors:** Szabolcs István Antal, Bálint Kincses, Dániel Veréb, András Király, Eszter Tóth, Bence Bozsik, Péter Faragó, Nikoletta Szabó, Krisztián Kocsis, Krisztina Bencsik, Péter Klivényi, Zsigmond Tamás Kincses

**Affiliations:** 1 Department of Radiology, Albert Szent-Györgyi Clinical Center, University of Szeged, Szeged, Hungary; 2 Department of Psychiatry, Albert Szent-Györgyi Clinical Center, University of Szeged, Szeged, Hungary; 3 Institute of Diagnostic and Interventional Radiology and Neuroradiology, University Hospital Essen, Essen, Germany; 4 Department of Neurology, Albert Szent-Györgyi Clinical Center, University of Szeged, Szeged, Hungary; 5 Department of Neurobiology, Care Sciences and Society, Karolinska Institutet, Stockholm, Sweden

Correction to: *Scientific Repots* 10.1038/s41598-023-31706-5, published online 05 April 2023

The Acknowledgements section in the original version of this Article was incomplete.

“The author (PF) was supported by an OTKA Grant (FK 135870). The author (ZTK) was supported by an OTKA Grant (K 139415).”

now reads:

“The author (SIA) was supported by the ÚNKP-22-3-SZTE-232 New National Excellence Program of the Ministry for Culture and Innovation from the source of the National Research, Development and Innovation Fund. The author (PF) was supported by an OTKA Grant (FK 135870). The author (ZTK) was supported by an OTKA Grant (K 139415).”

The original Article has been corrected.

